# Uterine torsion of 720 degrees in the third trimester of pregnancy and accompanying bladder torsion: a case report

**DOI:** 10.11604/pamj.2018.29.175.14101

**Published:** 2018-03-26

**Authors:** Cetin Kilicci, Ilhan Sanverdi, Evrim Bostanci, Cigdem Yayla Abide, Semra Kayatas Eser

**Affiliations:** 1 Zeynep Kamil Maternity and Children’s Training and Research Hospital, Department of Obstetrics and Gynecology, Istanbul, Turkey

**Keywords:** Uterine torsion, uterine atony, bladder torsion

## Abstract

Partial rotation of the uterus not more than 45 degrees to the right is considered to be normal. Since all cases are not reported, the incidence of uterine torsion in pregnancy is not known exactly. In the literature, there have been reports of cases with uterine torsion ranging from 45 to 720 degrees. This is a case report of uterine torsion of 720 degrees with accompanying bladder torsion, developing after two caesarean sections, and developing of uterine atony after the operation.

## Introduction

Partial rotation of gravid uterus not more than 45 degrees to the right is considered to be normal due to the close association between the sigmoid colon and the uterus. Uterine torsion in excess of 45 degrees is rare in the practice of obstetrics. However, it is a sufficient condition to cause serious morbidity and mortality for both mother and baby [1]. Clinical symptoms are either absent or nonspecific. Diagnosis is usually made by laparotomy. Since all cases are not reported, the incidence of uterine torsion in pregnancy is not known exactly. Don Wilson et al. indicated that 212 cases have been reported in the literature until 2006 [2]. In this article, a case of uterine torsion of 720 degrees accompanied by bladder torsion at 37 weeks of gestation was presented.

## Patient and observation

A 38-year-old woman (height, 165 cm; weight, 65 kg; gravida 4; para 2; abortus 1) with a history of two caesarean sections (CS), 37 weeks of gestation was referred to Perinatology clinic for the purpose of blood sugar regulation with a prediagnosis of non-regulated gestational diabetes (GDM), breech presentation, and polyhydramnios. She was hemodynamically stable and afebrile, the uterus was soft. Cardiotocography was reassuring and the blood glucose levels were regulated. After 48 hours, severe abdominal pain, severe vomiting, and loss of consciousness were developed. A fetal heart rate of < 60 bpm was determined on obstetric ultrasound (US). A CS was performed for suspected placental abruption as a consequence of worsening of abdominal pain.

**Figure 1:**
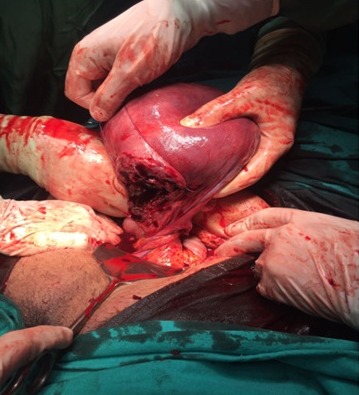
720-degree uterine torsion with the bladder

**Figure 2:**
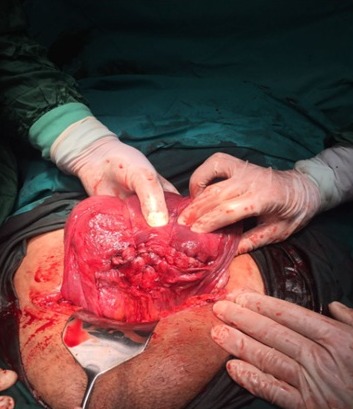
B-lynch sutures were performed for atony hemorrhage after detorsion

A laparotomy was performed through a transverse abdominal incision. Istmo-cervical torsion of the uterus including also fundus of the urinary bladder with 720 degrees was observed. Detorsion could not be performed. The peritoneal covering of the urinary bladder was observed over the region of uterine torsion. Hysterotomy was performed via a transverse uterine incision and a male baby weighing 3050 g was delivered (Figure 1). A 720 degrees derotation of the uterus was performed. B-Lynch compression sutures were performed due to bleeding and uterine atony. Since standard surgical procedures had failed to stop bleeding, bilateral hypogastric artery ligation was performed (Figure 2). The bleeding was controlled. The operation took 80 minutes. The patient received a postoperative transfusion of erythrocyte suspension. The baby was followed up for 2 days in the newborn intensive care unit postoperatively due to respiratory distress. The patient was discharged with her baby on the 3^rd^ postoperative day.

## Discussion

Uterine torsion is defined as the rotation of the uterus more than 45 degrees around the long axis. In the literature, there are reported cases of uterine torsion ranging from 45 degrees to 720 degrees [3]. The incidence of uterine torsion is quite low. It causes serious maternal and fetal morbidity and mortality approximately with a rate of 12-18% [2]. The cause of uterine torsion is not known. Uterine torsion may develop at every maternal age and gestation weeks during pregnancy without an underlying cause [1]. Uterine fibroids, adnexal masses, uterine anomalies, fetal presentation anomalies, polyhydramnios, maternal connective tissue elaxity have been reported to cause uterine torsion [4, 5]. In our case; polyhydramnios associated with GDM and presence of breech presentation might be risk factors for uterine torsion.

Clinical symptoms of uterine torsion in pregnancies may be variable. It can be asymptomatic, or it can result in a severe acute abdomen that can cause fetal and maternal mortality. Because of the presence of fetal bradycardia and acute abdominal pain, ablation placenta was considered as the first condition in this case. Ultrasonography can be used in the diagnosis of uterine torsion. Changes in known placental localization, loss of flow in the uterine artery dopplers, and abnormal localization of the ovary can be detected at ultrasonography [6]. In situations where the immediate operation is not required, the x-shaped configuration on the bladder may be indicated by MRI [7]. As in our case; most cases are diagnosed during the operation.

It is important to determine the degree of torsion to avoid bladder injury and ureter injury at the back of the uterus during operations where detorsion cannot be performed. In such cases, the baby can be delivered in three ways: vertical uterine fundal incision, uterine posterior wall incision or high transverse incision from the anterior wall of the uterus by observing and protecting the bladder plication [8]. In our case, the uterus could not be detorsioned due to advanced degrees of torsion. We delivered the fetus with a high transverse incision by observing and protecting the bladder plication. In order to reduce the recurrence rate of uterine torsion; round ligament plication was recommended in the early postpartum period [9] and uterosacral ligament plication was recommended in the late postpartum period [10]. Although we did not perform any plication in our case, we observed no early postpartum complications.

## Conclusion

To the best of our knowledge, this is the first case with regards to uterine torsion with accompanying bladder torsion, developing after two caesarean sections, and developing of uterine atony after the operation. Uterine torsion should be kept in mind in the cases of acute abdominal pain. It is important to determine the degree of torsion and place of the uterine incision (whether the anterior wall or the posterior wall of the uterus) for avoiding complications.

## Competing interests

The authors declare no competing interests.
